# PRISMA-S: an extension to the PRISMA Statement for Reporting Literature Searches in Systematic Reviews

**DOI:** 10.1186/s13643-020-01542-z

**Published:** 2021-01-26

**Authors:** Melissa L. Rethlefsen, Shona Kirtley, Siw Waffenschmidt, Ana Patricia Ayala, David Moher, Matthew J. Page, Jonathan B. Koffel, PRISMA-S Group, Heather Blunt, Tara Brigham, Steven Chang, Justin Clark, Aislinn Conway, Rachel Couban, Shelley de Kock, Kelly Farrah, Paul Fehrmann, Margaret Foster, Susan A. Fowler, Julie Glanville, Elizabeth Harris, Lilian Hoffecker, Jaana Isojarvi, David Kaunelis, Hans Ket, Paul Levay, Jennifer Lyon, Jessie McGowan, M. Hassan Murad, Joey Nicholson, Virginia Pannabecker, Robin Paynter, Rachel Pinotti, Amanda Ross-White, Margaret Sampson, Tracy Shields, Adrienne Stevens, Anthea Sutton, Elizabeth Weinfurter, Kath Wright, Sarah Young

**Affiliations:** 1 Health Science Center Libraries, George A. Smathers Libraries, University of Florida, Gainesville, USA; 2 UK EQUATOR Centre, Centre for Statistics in Medicine (CSM), Nuffield Department of Orthopaedics, Rheumatology and Musculoskeletal Sciences (NDORMS), Botnar Research Centre, University of Oxford, Windmill Road, Oxford, OX3 7LD UK; 3 Institute for Quality and Efficiency in Health Care, Cologne, Germany; 4 Gerstein Science Information Centre, University of Toronto, Toronto, Canada; 5 Centre for Journalology, Clinical Epidemiology Program, Ottawa Hospital Research Institute, The Ottawa Hospital, General Campus, Centre for Practice Changing Research Building, 501 Smyth Road, PO BOX 201B, Ottawa, Ontario K1H 8L6 Canada; 6 School of Public Health and Preventive Medicine, Monash University, Melbourne, Australia; 7 University of Minnesota, Minneapolis, USA

**Keywords:** Systematic reviews, Reporting guidelines, Search strategies, Literature search, Information retrieval, Reproducibility

## Abstract

**Background:**

Literature searches underlie the foundations of systematic reviews and related review types. Yet, the literature searching component of systematic reviews and related review types is often poorly reported. Guidance for literature search reporting has been diverse, and, in many cases, does not offer enough detail to authors who need more specific information about reporting search methods and information sources in a clear, reproducible way. This document presents the PRISMA-S (Preferred Reporting Items for Systematic reviews and Meta-Analyses literature search extension) checklist, and explanation and elaboration.

**Methods:**

The checklist was developed using a 3-stage Delphi survey process, followed by a consensus conference and public review process.

**Results:**

The final checklist includes 16 reporting items, each of which is detailed with exemplar reporting and rationale.

**Conclusions:**

The intent of PRISMA-S is to complement the PRISMA Statement and its extensions by providing a checklist that could be used by interdisciplinary authors, editors, and peer reviewers to verify that each component of a search is completely reported and therefore reproducible.

**Supplementary Information:**

The online version contains supplementary material available at 10.1186/s13643-020-01542-z.

## Introduction

One crucial component of a systematic review is the literature search. The literature search, or information retrieval process, not only informs the results of a systematic review; it is the underlying process that establishes the data available for analysis. Additional components of the systematic review process such as screening, data extraction, and qualitative or quantitative synthesis procedures are dependent on the identification of eligible studies. As such, the literature search must be designed to be both robust and reproducible to ensure the minimization of bias.

Guidelines exist for both the conduct of *literature searches* (Table 2) for systematic reviews and their reporting [2–7]. Problematically, however, the many guidelines for reporting systematic review searches share few common reporting elements. In fact, Sampson et al. discovered that of the eleven instruments designed to help authors report literature searches well, only one item appeared in all eleven instruments [8]. Though Sampson et al.’s study was conducted in 2007, the problem has only been compounded as new checklists and tools have continued to be developed. The most commonly used reporting guidance for systematic reviews, which covers the literature search component, is the Preferred Reporting Items for Systematic reviews and Meta-Analyses Statement, or PRISMA Statement [9]. The 2009 PRISMA Statement checklist included three items related to literature search reporting, items 7, 8, and 17:

Item 7: Describe all information sources (e.g., databases with dates of coverage, contact with study authors to identify additional studies) in the search and date last searched.

Item 8: Present full electronic search strategy for at least one database, including any limits used, such that it could be repeated.

Item 17: Give numbers of studies screened, assessed for eligibility, and included in the review, with reasons for exclusions at each stage, ideally with a flow diagram.

Despite wide usage of the PRISMA Statement [10], compliance with its items regarding literature search reporting is low [11–14]. Even for those studies which explicitly reference PRISMA, there is only slight, statistically non-significant evidence of improved reporting, as found by Page et al. [15]. Part of the challenge may be the multifactorial nature of each of the PRISMA items relating to searches; authors may feel if they completed one of the components of the item that they can check off that item altogether. Another part of the challenge may be that many systematic reviews do not include librarians or information specialists as members of the systematic review team or as authors on the final manuscript [11, 16–18]. Preliminary research suggests that librarian or information specialist involvement is correlated with reproducibility of searches [16–18], likely due to their expertise surrounding search development and documentation. However, reviews where librarians are authors still include reproducible searches only 64% of the time [17].

A larger issue may be that, even amongst librarians and information specialists, debate exists as to what constitutes a reproducible search and how best to report the details of the search. Researchers assessing the reproducibility of the search have used varying methods to determine what constitutes a reproducible search [11, 17, 19, 20]. Post-publication peer review of search methods, even amongst Cochrane reviews, which generally have superior reporting compared to non-Cochrane reviews [15], has shown that reporting that appears complete may still pose challenges for those wishing to reproduce searches [20–24]. Furthermore, little guidance on how to report searches using information sources or methods other than literature databases, such as searching web sites or study registries, exists [25, 26].

Incomplete reporting of the literature search methods can introduce doubt and diminish trust in the final systematic review conclusions. If researchers are unable to understand or reproduce how information was gathered for a systematic review, they may suspect the authors of having introduced bias into their review by not conducting a thorough or pre-specified literature search. After observing the high number of systematic reviews with poorly reported literature searches, we sought to create an extension to the PRISMA statement. Our aims were four-fold:

1. To provide extensive guidance on reporting the literature search components of a systematic review.
2. To create a checklist that could be used by authors, editors, and peer reviewers to verify that each component of a search was completely reported and therefore reproducible.
3. To develop an interdisciplinary checklist applicable to all method-driven literature searches for evidence synthesis.
4. To complement the PRISMA Statement and its extensions.

Because we intend the checklist to be used in all fields and disciplines, we use “systematic reviews” throughout this document as a representative name for the entire family of evidence syntheses [27]. This includes, but is not limited to, scoping reviews, rapid reviews, realist reviews, metanarrative reviews, mixed methods reviews, umbrella reviews, and evidence maps [28]. We use the term “literature search” or “search” throughout to encompass the full range of possible search methods and information sources.

## Part 1: Developing the Checklist

After consultation with members of the PRISMA Statement steering group (D.M. and D.G.A.), we formed an executive committee (M.L.R, J.K., S.K.) and developed a protocol [29] according to the steps outlined in the “Guidance for Developers of Health Research Reporting Guidelines [30].” The protocol was registered on the EQUATOR Network [29]. We identified 405 potential items relevant to reporting searches in systematic reviews from 61 sources (see Additional file 1) located through a search of MEDLINE via Ovid, Embase via Embase.com, and LISTA via EBSCOhost, in addition to reviewing all of the sources identified by the EQUATOR Network relating to systematic reviews. We also searched our personal files and examined references of included documents for additional sources. Details of the search are available in Additional file 1. Sources included both explicit reporting guidelines and studies assessing reproducibility of search strategies. The 405 items were reviewed for overlap and consolidated into 123 remaining items for potential inclusion in a checklist.

To narrow the list into a usable checklist, we then used a three-step Delphi survey process [31]. The first survey included the initially identified 123 items and asked respondents to rate each item on a 4-point Likert-type scale. Items that 70% of experts rated as 3 or 4 (4 being “essential” and 1 “not important”) and that received a mean score of at least 3.25 were retained for rating in the second round of the Delphi process. Respondents to the first survey were invited to participate in the second and third rounds. The second round asked respondents to pick the 25 most essential items out of the remaining 53 potential items; the third round was identical, except respondents also selected the most appropriate location for reporting their selected items (e.g., in the main text, or a supplementary file). The items were ranked and categorized by general theme for discussion at an in-person consensus conference.

We created a list of one hundred and sixty-three international experts, including librarian and information specialists with expertise in systematic reviews, researchers who had written about systematic review reporting, journal editors, and systematic review methodologists, to whom we sent our initial Delphi survey. The list of experts was created using a combination of publications, mailing lists, conference proceedings, and knowledge of the authors to represent research groups and experts in 23 countries. We received 52 responses (32% response rate) to the first survey, and of these, 35 (67% response rate) completed both surveys two and three. This study was declared exempt by the University of Utah Institutional Review Board (IRB_00088425).

The results of the Delphi process were reported at a consensus conference meeting that took place in May 2016 concurrently with Mosaic ‘16, the joint meeting of the Medical Library Association, Canadian Health Libraries Association/Association des bibliothèques de la santé du Canada, and the International Clinical Librarian Conference (ICLC). 38 individuals attended the consensus conference, 14 (37%) of whom had participated in the Delphi surveys. At the consensus conference, the grouped and ranked remaining items were distributed to small groups who were asked to discuss, consolidate, remove, or add missing critical items under the guidance of a group leader. After two rounds of discussion, the group leaders presented the discussion and proposed list items from their small groups for consideration by the whole group of experts.

Upon completion of the consensus conference, 30 items remained from those identified during the Delphi process, with an additional three items that had been excluded during the Delphi process added back to the draft checklist because meeting attendees considered them critical to the guideline. The list was then consolidated and reviewed by executive committee members, including two new information specialist members (S.W. and A.P.A). The draft checklist and explanation and elaboration document was released to the public on March 20, 2019, along with all data and study materials [32]. All participants in the Delphi process and/or consensus conference were contacted via email with instructions on how to provide feedback on the draft checklist items and/or elaboration and explanation document by commenting directly on the explanation and elaboration draft using a private commenting system, Hypothesis [33], or if preferred, via email. Comments from other interested individuals were solicited via Twitter, conference presentations, and personal contacts. Comments were collected from the private Hypothesis group, the public Hypothesis comments, and via email. All comments were combined into a single document. Executive committee members reviewed each comment in duplicate to indicate what type of feedback was received (i.e., linguistic, major substantive, minor substantive, or unclear) and, for substantive comments, whether change was recommended or required further discussion.

During the draft and revision process (March 20–June 15, 2019), 358 separate comments were received from 22 individuals and organizations. Based upon the extensive feedback received, the executive team revised the checklist and developed the next iteration, which was released on December 6, 2019, to coincide with the 2019 Virtual Cochrane Colloquium Santiago. Additional feedback from this release was incorporated into the final checklist. Throughout the draft and revision process, several teleconferences were held with the lead of the PRISMA 2020 statement (M.J.P), as an update of the 2009 PRISMA statement was in development, to ensure that the content on search methods was consistent between the PRISMA 2020 and PRISMA-S guidelines [34, 35].

## Part 2: Checklist

PRISMA-S is a 16-item checklist that covers multiple aspects of the search process for systematic reviews. It is intended to guide reporting, not conduct, of the search. The checklist should be read in conjunction with the Explanation and Elaboration (Part 3), which provides more detail about each item. We also include two boxes, one a glossary of terms (see Table 2) and the other, guidance on depositing search data and method descriptions in online repositories (see Table 3).

The Explanation and Elaboration also includes examples of good reporting for each item. Each exemplar is drawn from published systematic reviews. For clarity, some exemplars are edited to match the style of this document, including any original citations, and abbreviations are spelled out to aid comprehension. Any other edits to the text are noted with square brackets. A description of the rationale behind the item is explained, followed by additional suggestions for clear reporting and a suggested location(s) for reporting the item.

Not every systematic review will make use of all of the items in the Information Sources and Methods section of the checklist, depending on the research question and the methods chosen by the authors. The checklist provides a framework for the current most common and recommended types of information sources and methods for systematic reviews, but authors should use and report those items relevant and appropriate to their review. The checklist may also be used for systematic review protocols to fully document the planned search, in conjunction with the PRISMA-P reporting guideline [36] (Table 1).

**Table 1:** PRISMA-S checklist. A downloadable version of the checklist is available on the PRISMA website [37]

SECTION/TOPIC	ITEM #	CHECKLIST ITEM
**INFORMATION SOURCES AND METHODS**		
Database name	1	Name each individual database searched, stating the platform for each.
Multi-database searching	2	If databases were searched simultaneously on a single platform, state the name of the platform, listing all of the databases searched.
Study registries	3	List any study registries searched.
Online resources and browsing	4	Describe any online or print source purposefully searched or browsed (e.g., tables of contents, print conference proceedings, web sites), and how this was done.
Citation searching	5	Indicate whether cited references or citing references were examined, and describe any methods used for locating cited/citing references (e.g., browsing reference lists, using a citation index, setting up email alerts for references citing included studies).
Contacts	6	Indicate whether additional studies or data were sought by contacting authors, experts, manufacturers, or others.
Other methods	7	Describe any additional information sources or search methods used.
**SEARCH STRATEGIES**		
Full search strategies	8	Include the search strategies for each database and information source, copied and pasted exactly as run.
Limits and restrictions	9	Specify that no limits were used, or describe any limits or restrictions applied to a search (e.g., date or time period, language, study design) and provide justification for their use.
Search filters	10	Indicate whether published search filters were used (as originally designed or modified), and if so, cite the filter(s) used.
Prior work	11	Indicate when search strategies from other literature reviews were adapted or reused for a substantive part or all of the search, citing the previous review(s).
Updates	12	Report the methods used to update the search(es) (e.g., rerunning searches, email alerts).
Dates of searches	13	For each search strategy, provide the date when the last search occurred.
**PEER REVIEW**		
Peer review	14	Describe any search peer review process.
**MANAGING RECORDS**		
Total records	15	Document the total number of records identified from each database and other information sources.
Deduplication	16	Describe the processes and any software used to deduplicate records from multiple database searches and other information sources.

## Part 3: Explanation and Elaboration

### Item 1. Database name

Name each individual database searched, stating the platform for each.

### Example

“The following electronic databases were searched: MEDLINE (Ovid), CINAHL (EBSCOhost), PsycINFO (Ovid), Cochrane Central Register of Controlled Trials (Ovid), SPORTDiscus (EBSCOhost), EMBASE (Ovid) and ProQuest Dissertations and Theses Global (ProQuest).” [38]

### Explanation

Databases are the most commonly used tool to locate studies to include in systematic reviews and meta-analyses [6, 39]. There is no single database that is able to provide a complete and accurate list of all studies that meet systematic review criteria due to the differences in the articles included and the indexing methods used between *databases* (Table 2). These differences have led to recommendations that systematic review teams search multiple databases to maximize the likelihood of finding relevant studies [6, 39, 40]. This may include using broad disciplinary databases (e.g., MEDLINE [41], Embase [42], Scopus [43]), specialized databases (e.g., PsycINFO [44] or EconLit [45]), or regional databases (e.g., LILACS [46] or African Index Medicus [47]).

**Table 2:** **Glossary**

**Browse**: Browsing is the practice of scanning for information by reviewing content. This may include using tables of contents, indices in books or other materials, web directories, full journal issues, specific web pages, or other types of information scanned without using a formal search strategy.	
**Citation index**: A type of database or database function that enables searchers to analyze relationships between publications through citations, including what publications have cited, and which publications are citing the publication(s) you are interested in. Common examples include Science Citation Index, Scopus, and Google Scholar.	
**Cited reference**: Publication referenced in a given publication.	
**Citing reference**: Publications that have referenced a given publication.	
**Database**: Within PRISMA-S, this refers to a literature database designed to search journal literature. Databases may be multidisciplinary or specialized. Many include specialized search features, subject headings, and structured data designed to facilitate easy and comprehensive searching. Examples include MEDLINE, EconLit, and PsycINFO.	
**Digital object identifier**: Also called a DOI, a digital object identifier is a unique code assigned to a publication, dataset, or other online item or collection that will remain constant over time.	
**Field code**: Unique to each database platform and database, field codes are used to specify where a term is searched for in a database record. In PubMed, for instance, the field code [tiab] is placed after a search term to tell the database to search only within the title and abstract fields.	
**Filter**: Filters are predefined combinations of search strategies designed to locate references meeting certain criteria, usually publication type, topic, age group, or other categorization. Filters generally are combinations of keywords, subject headings or thesaurus terms, logical operators, and database-specific syntax. Many filters are validated and offer sensitivity and specificity information that allows searchers to determine their usefulness for a given search. Filters may also be called hedges or optimal search strategies and are designed for other searchers to use and reuse.	
**Indexing**: Application of standard terminology to a reference to describe the contents of the full article. Depending on the database or other information source, indexers may add subject headings or thesaurus terms as well as list age groups, language, human studies, study design, publication type, or other descriptive terms. Examples of indexing terminology include MEDLINE’s Medical Subject Headings (MeSH) and Embase’s EMTREE.	
**Information source:** Any database or other resource such as a web site, journal table of contents, email alert, web directory, contact with authors or industry, study registry, or preprint server, etc. searched or browsed as part of the search.	
**Literature search**: Here, an overall term for the entire information retrieval process as part of a systematic review. This includes the full range of searching methods and information sources, including databases, study registries, regulatory datasets, web searches, government documents, unpublished data, and much more.	
**Limits**: Features built into databases to allow searchers to quickly restrict their search by one or more categories. Common limits built into databases include publication date ranges, language, gender, age group, and publication type. Limits are different from filters (see above) and are also not the inclusion/exclusion criteria used in the screening process.	
**Multi-database search**: Many database platforms offer more than one database on the same platform. Some platforms allow users to search these multiple databases at one time, for example using the Ovid platform to simultaneously search MEDLINE, Embase, and the Cochrane Database of Systematic Reviews.	
**Peer review**: In PRISMA-S, this refers to the peer review of search strategies prior to executing the search. Peer review is used to identify errors, missing keywords or subject headings, and other issues within a search strategy. One commonly used tool for search strategy peer review is the Peer Review of Electronic Search Strategies (PRESS) Guideline [1].	
**Platform**: Many databases are available on multiple different systems, each of which have their own specifications for how a search strategy can be constructed. The location or host system of the database is the platform. Platform is sometimes referred to as the interface or vendor. Common examples include Ovid, EBSCOhost, ProQuest, and Web of Science.	
**Records:** Individual items retrieved from any type of search, though most commonly used in conjunction with database searches. Records may also be referred to as references or hits.	
**Repository**: An online archive for varying types of electronic files, including text documents, data files, and more. Repositories may be hosted by an institution or more broadly available.	
**Rerun**: Re-executing the same search strategy in the same database one or more times after the original search was conducted. See *Updating a search strategy*.	
**Search**: Overall term for the entire information retrieval process as part of a systematic review. It can also refer to searching a specific database, web site, or other information source.	
**Search strategy**: Structure of terms, logical operators, and syntax elements (field codes (see above), adjacency operators, phrases, etc) that is used to search a database or other information source. A search strategy may be very simple or very complex, depending on the information source and requirements of the search.	
**Sensitivity:** A measure of how well a search strategy finds relevant articles, sensitivity (usually expressed as a percentage) is the number of relevant records found with a search strategy divided by the total number of relevant records in a given information source. Highly sensitive search strategies or filters detect most or all records that are relevant. Together with specificity, sensitivity is a measure used to assess the performance of filters. Sensitivity may also be called recall.	
**Specificity:** A measure of how well a search strategy omits irrelevant articles, specificity (usually expressed as a percentage) is the number of irrelevant records not found with (or excluded by) a search strategy divided by the total number of irrelevant records in a given information source. Search strategies or filters with high specificity will find few irrelevant articles. Together with sensitivity, specificity is often used to assess the performance of filters.	
**Study registry**: A database of records of research studies in progress. Originally designed for clinical trials as a location for patients to find clinical trials to join, study registries have spread beyond biomedical research to other fields. Study registries may also contain research results, posted after study completion.	
**Supplementary materials**: Additional content for a study that does not fit in the main manuscript text. For a systematic review, supplementary materials should include full search strategies for all information sources and more complete search methods description. Supplementary materials are generally submitted with the manuscript for peer review.	
**Syntax:** Search structure and organization, based on a set of rules governing how a search operates in a specific database and platform. Rules might include field codes, phrase and adjacency searching, Boolean operators, and truncation, amongst others.	
**Systematic review**: For the purposes of PRISMA-S, systematic review is used for the entire family of methods-based reviews. This includes rapid reviews, scoping reviews, meta-narrative reviews, realist reviews, meta-ethnography, and more.	
**Updating a search strategy**: To ensure currency, authors often search for additional information throughout the systematic review process or before submitting a report. The search may be updated by running the exact same search (rerunning the search) or by conducting a new or modified search to locate additional references.	

Many of these literature databases are available through multiple different search *platforms* (Table 2). For example, the MEDLINE database is available through at least 10 different platforms, including Ovid, EBSCOhost, Web of Science, and PubMed. Each platform offers different ways of searching the databases, such as platform-specific *field codes* (Table 2), phrase searching, truncation, or searching full-text versus abstract and keyword only [48]. Different platforms may contain additional data that are not available in the original database, such as times cited, social media impact, or additional keywords. These differences between the platforms can have a meaningful impact on the results provided [48–50].

Authors should identify which specific literature databases were searched to locate studies included in the systematic review. It is important that authors indicate not only the database, but the platform through which the database was searched. This helps readers to evaluate the quality and comprehensiveness of the search and supports reproducibility and *updating* (Table 2) in the future by allowing the strategy to be copied and pasted as recommended in Item 8, below.

The distinctions between database and platform may not always be clear to authors, especially when the database is the only one available through a platform (e.g., Scopus [43]). In these cases, authors may choose to include the web address of the database in the text or the bibliography to provide clarity for their readers.

#### Suggested location for reporting

Report each database name and platform in the methods section and any *supplementary materials* (Table 2). If space permits, report key database names in the abstract.

### Item 2. Multi-database searching

If databases were searched simultaneously on a single platform, state the name of the platform, listing all of the databases searched.

### Examples

“The MEDLINE and Embase strategies were run simultaneously as a multi-file search in Ovid and the results de-duplicated using the Ovid de-duplication tool.” [51]

“A systematic literature search was performed in Web of Knowledge™ (including KCI Korean Journal Database, MEDLINE, Russian Science Citation Index, and SciELO Citation Index)….” [52]

### Explanation

Authors may choose to search multiple databases at once through a single search platform to increase efficiency. Along with the name of the platform, it is necessary to list the names of each of the individual databases included as part of the search. Including information about using this approach in the text of the manuscript helps readers immediately understand how the search was constructed and executed. This helps readers determine how effective the *search strategy* (Table 2) will be for each database [1].

#### Suggested location for reporting

Report any *multi-database search* (Table 2) in the methods section and any supplementary materials. If space permits, report key individual database names in the abstract, even if run through a multi-database search.

### Item 3. Study registries

List any study registries searched.

### Example

“[We] searched several clinical trial registries (ClinicalTrials.gov, Current Controlled Trials (www.controlled-trials.com), Australian New Zealand Clinical Trials Registry (www.actr.org.au), and University Hospital Medical Information Network Clinical Trials Registry (www.umin.ac.jp/ctr)) to identify ongoing trials.” [53]

### Explanation

Study registries are a key source of information for systematic reviews and meta-analyses in the health sciences and increasingly in other disciplines. In the health sciences, *study registries* (Table 2) allow researchers to locate ongoing clinical trials and studies that may have gone unpublished [54–56]. Some funders, including the National Institutes of Health, require principal investigators to share their data on study registries within a certain time frame after grant completion [57]. This data may not have been published in any other location, making study registries a critical component of an information strategy, though timely reporting remains a challenge [58, 59]. Different countries have their own study registries, as do many pharmaceutical companies.

Outside the health sciences, study registries are becoming increasingly important as many disciplines adopt study pre-registration as a tactic for improving the rigor of research. Though not yet as established as in the health sciences, these study registries are continually expanding and will serve as key sources for finding unpublished studies in fields in the social sciences and beyond.

To fully describe the study registries searched, list the name of each study registry searched, and include a citation or link to the study registry.

#### Suggested location for reporting

Report any study registries searched in the methods section and any supplementary materials.

### Item 4. Online resources and browsing

Describe any online or print source purposefully searched or browsed (e.g., tables of contents, print conference proceedings, web sites), and how this was done.

### Examples

*“*We also searched the grey literature using the search string: “public attitudes” AND “sharing” AND “health data” on Google (in June 2017). The first 20 results were selected and screened.” [60]

“The grey literature search was conducted in October 2015 and included targeted, iterative hand searching of 22 government and/or research organization websites that were suggested during the expert consultation and are listed in S1 Protocol. Twenty two additional citations were added to the review from the grey literature search.” [61]

“To locate unpublished studies, we searched Embase [via Embase.com] for conference proceedings since 2000 and hand-searched meeting abstracts of the Canadian Conference on Physician Health and the International Conference on Physician Health (2012 to 2016).” [62]

### Explanation

Systematic reviews were developed to remove as much bias as possible from the literature review process. One of the most important ways they achieve this reduction in bias is by searching beyond literature databases, which are skewed towards English-language publications with positive results [63, 64]. To achieve a fuller picture of what the research on a specific topic looks like, systematic reviewers could seek out research that may be in progress and research that was never published [6]. Using other methods of finding research also helps identify research that may have been indexed in literature databases, but went undiscovered when searching those sources [40]. Seeking out this research often involves a complex strategy, drawing on a wealth of online and print resources as well as personal contacts.

#### Web search engines and specific web sites

Searching general internet search engines and searching the contents of specific websites is a key component of many systematic reviews [26, 65]. Government, non-profit organization, and pharmaceutical company websites, for example, contain a wealth of information not published elsewhere [6, 66]. Though searching a general search engine like Google or using a general search engine to search a specific website may introduce some bias into the search methodology through the personalization algorithms inherent in many of these tools [67, 68], it is still important to fully document how web searches were conducted [65].

Authors should list all websites searched, along with their corresponding web address. Readers should be able to clearly understand if researchers used a website’s native search interface or advanced search techniques within a general search engine. If authors used a general search engine, authors should declare whether steps were taken to reduce personalization bias (e.g., using “incognito” mode in a browser). Authors may choose whether to detail the websites searched within the text (i.e., Google (http://www.google.com)), by citing the websites in the bibliography, or by listing the website with corresponding web address in supplementary material, as shown in the examples above.

Review teams may occasionally set an artificial limit to the number of items they will screen from a given search or source [65]. This is because searching web search engines and individual websites will often lead to an unmanageable number of results, the search engine itself may only display a restricted number of results (e.g., Google will only display 1000 results), or the team has a finite budget or timeline to complete the review. Thus, many systematic review teams utilizing web search engines will often pre-designate a limit to the number of results they review. If review teams choose to review a limited set of results, it should be noted in the text, along with the rationale.

#### Conference proceedings

Studies show that large percentages of research presented as papers and posters at conferences never make their way into the published literature, particularly if the study’s results were statistically negative [63, 69]. Conference proceedings are often the only way to locate these studies. Including conference proceedings in a systematic review search helps minimize bias [70]. The introduction of online conference proceedings has been a boon to researchers and reduced the need to review printed abstract books. Additionally, some databases either include conference proceedings along with journal articles (i.e., Embase [42]) or contain only conference proceedings (i.e., ProceedingsFirst [71] or Directory of Published Papers [72]). Some conferences have made their abstracts available in a single database (i.e., International AIDS Society’s Abstract Archive [73]). When using these types of databases to search conference proceedings, authors can treat them as above in Item 1.

Individual conferences’ online proceedings may be password-protected for association members or conference attendees [74]. When reporting on conference proceedings searched or *browsed* (Table 2) via a conference or association’s online or print proceedings, authors must specify the conference names, the dates of conferences included, and the method used to search the proceedings (i.e., browsing print abstract books or using an online source). If the conference proceedings are searched online, authors should specify the web address(es) for the conference proceedings and the date(s) of the conferences. If the conference proceedings are published in a journal, the authors should cite the journal. If the proceedings are a standalone publication, authors may choose to cite them using the same methods used to cite a book or by providing the full information about the conference (name, location, dates, etc.) in a supplementary file.

#### General browsing

Authors also commonly browse print or online tables of contents, full contents of journals, or other sources that are the most likely to contain research on the topic sought. When purposefully browsing, describe any method used, the name of the journal or other source, and the time frame covered by the search, if applicable.

#### Suggested location for reporting

Report online *information sources* (Table 2) searched or browsed in the methods section and in any supplementary materials. Systematic reviews using several of these methods, or using multiple information sources for each method, may need to report their methods briefly in the methods section, but should fully report all necessary information to describe their approaches in supplementary materials.

### Item 5. Citation searching

Indicate whether cited references or citing references were examined, and describe any methods used for locating cited/citing references (e.g., browsing reference lists, using a citation index, setting up email alerts for references citing included studies).

### Examples

“Reference lists of included articles were manually screened to identify additional studies.” [75]

“[W]e used all shared decision making measurement instruments that were identified in Gärtner et al’s recent systematic review (Appendix A). We then performed a systematic citation search, collecting all articles that cited the original papers reporting on the development, validation, or translation of any the observational and/or self-reported shared decision making measurement instruments identified in that review. An experienced librarian (P.J.E.) searched Web of Science [Science Citation Index] and Scopus for articles published between January 2012 and February 2018.” [76]

“We [conducted] citation tracking of included studies in Web of Science Core Collection on an ongoing basis, using citation alerts in Web of Science Core Collection.” [77]

### Explanation

One of the most common search methods is reviewing the references or bibliographies of included studies [11, 17]. This type of citation searching (looking for cited references) can be additive to other cited reference searching methods, such as examining bibliographies of relevant systematic reviews. In addition, researchers may choose to look for articles that cite specified studies [78]. This may include looking beyond one level forwards and backwards (e.g., examining the bibliographies of articles cited by specified articles) [78]. Looking at bibliographies of included articles or other specified articles is often conducted by examining full-text articles, but it can also be accomplished using online tools called *citation indexes* (Table 2).

The use of these methods can be complicated to describe, but the explanation should clearly state the database used, if applicable (i.e., Scopus, Google Scholar, Science Citation Index) and describe any other methods used. Authors also must cite the “base” article(s) that citation searching was performed upon, either for examining *cited* or *citing* articles (Table 2). If the same database is used for both a topical search as well as citation searching, describe each use separately. For manually checking the reference lists for included articles, a simple statement as in the first example is sufficient.

#### Suggested location for reporting

Report citation searching details in the methods section and in any supplementary materials.

### Item 6. Contacts

Indicate whether additional studies or data were sought by contacting authors, experts, manufacturers, or others.

### Examples

“We contacted representatives from the manufacturers of erythropoietin-receptor agonists (Amgen, Ortho-Biotech, Roche), corresponding or first authors of all included trials and subject-area experts for information about ongoing studies.” [79]

“We also sought data via expert requests. We requested data on the epidemiology of injecting drug use and blood-borne viruses in October, 2016, via an email distribution process and social media. This process consisted of initial emails sent to more than 2000 key experts and organisations, including contacts in the global, regional, and country offices of WHO, UNAIDS, Global Fund, and UNODC (appendix p 61). Staff in those agencies also forwarded the request to their colleagues and other relevant contacts. One member of the research team (SL) posted a request for data on Twitter, which was delivered to 5525 individual feeds (appendix p 62).” [80]

### Explanation

Contacting manufacturers (e.g., pharmaceutical companies), or reaching out to authors or experts directly or through organizations, is a key method to locate unpublished and ongoing studies [6]. Contacting authors or manufacturers may also be necessary when publications, conference proceedings, or clinical trials registry records do not provide the complete information needed [63, 81]. Contacting manufacturers or regulating agencies might be required to acquire complete trial data from the clinical study reports [82, 83]. More broad calls for evidence may also be conducted when no specific groups or individuals are targeted.

Contact methods may vary widely, depending on the context, and may include personal contact, web forms, email mailing lists, mailed letters, social media contacts, or other methods. As these strategies are inherently difficult to reproduce, researchers should attempt to give as much detail as possible on what data or information was sought, who or what group(s) provided data or information, and how the individuals or groups were identified.

#### Suggested location for reporting

Report information about contacts to solicit additional information in the methods section and in any supplementary materials. Systematic reviews using elaborate calls for evidence or making extensive use of contacts as an information source may need to report their methods briefly in the methods section, but should fully report all necessary information to describe their approaches in supplementary materials.

### Item 7. Other methods

Describe any additional information sources or search methods used.

### Examples

“We also searched… our personal files.” [84]

“PubMed’s related articles search was performed on all included articles.” [85]

A thorough systematic review may utilize many additional methods of locating studies beyond database searching, many of which may not be reproducible methods. A key example is searching personal files. Another is using databases’ built in tools, such as PubMed’s Related Articles feature [86] or Clarivate Analytics’ Web of Science’s Related Records feature [87], to locate relevant articles based on commonalities with a starting article. Because these tools are often proprietary and their algorithms opaque, researchers may not be able to replicate the exact results at a later date. To attempt to be as transparent as possible, researchers should both note the tool that was used and cite any articles these operations were run upon. For all “other” methods, it is still important to declare that the method was used, even if it may not be fully replicable.

#### Suggested location for reporting

Report information about any other additional information sources or search methods used in the methods section and in any supplementary materials.

### Item 8. Full search strategies

Include the search strategies for each database and information source, copied and pasted exactly as run.

### Examples

*Database search. Methods section description*. “The reproducible searches for all databases are available at DOI:10.7302/Z2VH5M1H.” [88]

*Database search. One of the full search strategies from supplemental materials in online repository*. “Embase.com (692 on Jan 19, 2017)

1. 'social media'/exp OR (social NEAR/2 (media* OR medium* OR network*)):ti OR twitter:ti OR youtube:ti OR facebook:ti OR linkedin:ti OR pinterest:ti OR microblog*:ti OR blog:ti OR blogging:ti OR tweeting:ti OR 'web 2.0':ti
2. 'professionalism'/exp OR 'ethics'/exp OR 'professional standard'/de OR 'professional misconduct'/de OR ethic*:ab,ti OR unprofessional*:ab,ti OR professionalism:ab,ti OR (professional* NEAR/3 (standard* OR misconduct)):ab,ti OR ((professional OR responsib*) NEAR/3 (behavi* OR act OR conduct*)):ab,ti
3. #1 AND #2 AND [english]/lim NOT ('conference abstract':it OR 'conference paper':it) [88]

*Online resources and browsing. Methods section description*. “The approach to study identification from this systematic review is transparently reported in the Electronic Supplementary Material Appendix S1.” [89]

*Online resources and browsing. One of the full online resource search strategies reported in supplement*. “Date: 12/01/16. Portal/URL: Google. https://www.google.co.uk/webhp?hl=en. Search terms: ((Physical training) and (man or men or male or males) and (female or females or women or woman) and (military)). Notes: First 5 pages screened on title (n=50 records).” [89]

### Explanation

Systematic reviews and related review types rely on thorough and complex search strategies to identify literature on a given topic. The search strategies used to conduct this data gathering are essential to the transparency and reproducibility of any systematic review. Without being able to assess the quality of the search strategies used, readers are unable to assess the quality of the systematic review [9, 11, 17].

When space was at a premium in publications, complete reporting of search strategies was a challenge. Because it was necessary to balance the need for transparency with publication restrictions, previous PRISMA guidelines recommended including the complete search strategy from a minimum of one database searched [9]. Many systematic reviews therefore reported only the minimum necessary. However, reporting only selected search strategies can contribute to the observed irreproducibility of many systematic reviews [11, 17].

The prior versions of PRISMA did not elaborate on methods for reporting search strategies outside of literature databases. Subsequent to its publication, many groups have begun identifying the challenges of fully documenting other types of search methods [90, 91]. Now recommended is the explicit documentation of all of the details of all search strategies undertaken [91, 92]. These should be reported to ensure transparency and maximum reproducibility, including searches and purposeful browsing activities undertaken in web search engines, websites, conference proceeding databases, electronic journals, and study registries.

Journal restrictions vary, but many journals now allow authors to publish supplementary materials with their manuscripts. At minimum, all search strategies, including search strategies for web search engines, websites, conference proceedings databases, electronic journals, and study registries, should be submitted as a supplement for publication. Though most supplements are appropriately accessible on journal publishers’ web sites as submitted, supplements may disappear [17]. In addition, many supplements are only available to journal subscribers [93]. Similarly, manuscripts available on public access systems like PubMed Central [94] may not have the corresponding supplemental materials properly linked. For optimal accessibility, authors should upload complete documentation to a data *repository* (Table 2), an institutional repository, or other secure and permanent online archive instead of relying on journal publication (see Table 3 for additional information).

**Table 3:** Supplementary Materials

Fully documenting a search will require publication of supplementary materials. Due to the instability of supplementary materials published as part of a journal article, uploading complete documentation to a secure and permanent archive is recommended.	
**Options for secure and permanent archives** Many options exist for uploading documentation. Ideally, use an archive or repository that will provide a digital object identifier (DOI) for any uploaded materials (Table 2). These are a few of the many options available. *Institutional repository*: Many institutions or their affiliated libraries host online repository systems for their faculty, staff, and students. An example is the University of Michigan’s Deep Blue Data system (https://deepblue.lib.umich.edu/). *Open Science Framework* (http://osf.io/): The Open Science Framework (OSF) platform enables the storage of any documentation associated with a research study. It is possible to create DOIs for individual files or groups of files. OSF is openly and freely available. *figshare* (https://figshare.com/): figshare is a commercial platform that allows researchers to share any type of data or research output. It is possible to create DOIs for individual files or collections. *Zenodo* (https://zenodo.org/): Zenodo is a general purpose, freely available open access repository available from CERN for research data and associated materials. Uploaded materials are assigned DOIs.	
**What documentation to upload** Materials related to all PRISMA-S checklist items can be included in supplementary materials. Sufficient information should be uploaded that would enable an interested reader to replicate the search strategy. Specifically, it is recommended that documentation relating to the full search strategies for all information sources and methods be included in supplementary materials. Optionally, authors may wish to upload additional supplementary information, including files of all references retrieved, all references after deduplication, and all references to included studies. Authors who wish to share these files should note that abstracts are copyrighted materials and should be removed from files before sharing them publicly. For an example of supplementary materials related to a systematic review search, see: MacEachern, M. (2017). Literature search strategies for "Substance Use Education in Schools of Nursing: A Systematic Review of the Literature" [Data set]. University of Michigan - Deep Blue. 10.7302/Z24X560Q In this example, the materials shared include a Read Me file to explain the files, EndNote (.enlx) files of screened references, the original files imported into EndNote, and the complete, reproducible search strategies for all information sources.	

It is important to document and report the search strategy exactly as run, typically by copying and pasting the search strategy directly as entered into the search platform. This is to ensure that information such as the fields searched, term truncation, and combinations of terms (i.e., Boolean logic or phrases) are accurately recorded. Many times, the copied and pasted version of a search strategy will also include key information such as *limits* (see Item 9; Table 2) used, databases searched within a multi-database search, and other database-specific detail that will enable more accurate reporting and greater reproducibility. This documentation must also repeat the database or resource name, database platform or web address, and other details necessary to clearly describe the resource.

#### Suggested location for reporting

Report the full search strategy in supplementary materials as described above. Describe and link to the location of the supplementary materials in the methods section.

### Item 9: Limits and restrictions

Specify that no limits were used, or describe any limits or restrictions applied to a search (e.g., date or time period, language, study design) and provide justification for their use.

### Examples

*No limits*. “We imposed no language or other restrictions on any of the searches.” [95]

*Limits described without justification*. “The search was limited to the English language and to human studies.” [96]

“The following search limits were then applied: randomized clinical trials (RCTs) of humans 18 years or older, systematic reviews, and meta-analyses.” [97]

*Limits described with justification*. “The search was limited to publications from 2000 to 2018 given that more contemporary studies included patient cohorts that are most reflective of current co-morbidities and patient characteristics as a result of the evolving obesity epidemic.” [98]

*Limits described, one with justification*. “Excluded publication types were comments, editorials, patient education handouts, newspaper articles, biographies, autobiographies, and case reports. All languages were included in the search result; non-English results were removed during the review process…. To improve specificity, the updated search was limited to human participants.” [99]

### Explanation

Many databases have features that allow searchers to quickly restrict a search using limits. What limits are available in a database are unique to both the database and the platform used to search it. Limits are dependent on the accuracy of the indexer, the timeliness of indexing, and the quality of any publisher-supplied data. For instance, using database limits to restrict searches to randomized controlled trials will only find records identified by the indexer as randomized controlled trials. Since the indexing may take 6 months or more to complete for any given article, searchers risk missing new articles when using database limits.

Using database-provided limit features should not be confused with using *filters* (see Item 10; Table 2) or inclusion criteria for the systematic review. For example, systematic review teams may choose to only include English-language randomized controlled trials. This can be done using limits, a combination of a filter (see Item 10) and screening, or screening alone. It should be clear to the reader which approach is used. For instance, in the “*Limits described, with one justification*” example above, the authors used database limits to restrict their search by publication type, but they did not use database limits to restrict by language, even though that was a component of their eligibility criteria. They also used database limits to restrict to human participants in their search update.

It is important for transparency and reproducibility that any database limits applied when running the search are reported accurately, as their use has high potential for introducing bias into a search [1, 64, 100, 101]. Database limits are not recommended for use in systematic reviews, due to their fallibility [39, 100]. If used, review teams should include a statement of justification for each use of a database limit in the methods section, the limitations section, or both [102, 103]. In the examples above, only the last two examples provide some justification in the methods section (“to improve specificity” [99] and “contemporary studies included patient cohorts that are most reflective of current co-morbidities and patient characteristics as a result of the evolving obesity epidemic” [98]).

#### Suggested location for reporting

Report any limits or restrictions used or that no limits were used in the abstract, methods section, and in any supplementary materials, including the full search strategies (Item 8). Report the justification for any limits used within the methods section and/or in the limitations section.

### Item 10. Search filters

Indicate whether published search filters were used (as originally designed or modified), and if so, cite the filter(s) used.

### Example

“For our MEDLINE search we added a highly sensitive filter for identifying randomised trials developed by the Cochrane Collaboration [38]. For Embase we used the filter for randomised trials proposed by the Scottish Intercollegiate Guidelines Network [104].” [105]

### Explanation

Filters are a predefined combination of search terms developed to identify references with a specific content, such as a particular type of study design (e.g., randomized controlled trials) [106], populations (e.g., the elderly), or a topic (e.g., heart failure) [107]. They often consist of a combination of subject headings, free-text terms, and publication types [107]. For systematic reviews, filters are generally recommended for use instead of limits built into databases, as discussed in Item 9, because they provide the much higher *sensitivity* (Table 2) required for a comprehensive search [108].

Any filters used as part of the search strategy should be cited, whether published in a journal article or other source. This enables readers to assess the quality of the filter(s) used, as most published search filters are validated and/or peer reviewed [106, 107]. Many commonly used filters are published on the InterTASC Information Specialists’ Sub-Group [109], in the Cochrane Handbook [4, 39], and through the Health Information Research Unit of McMaster University [110].

#### Suggested location for reporting

Cite any search filter used in the methods section and describe adaptations made to any filter. Include the copied and pasted details of any search filter used or adapted for use as part of the full search strategy (Item 8).

### Item 11. Prior work

Indicate when search strategies from other literature reviews were adapted or reused for a substantive part or all of the search, citing the previous review(s).

### Example

“We included [search strategies] used in other systematic reviews for research design [111], setting [112, 113], physical activity and healthy eating [114–116], obesity [111], tobacco use prevention [117], and alcohol misuse [118]. We also used a search [strategy] for intervention (implementation strategies) that had been employed in previous Cochrane Reviews [119, 120], and which was originally developed based on common terms in implementation and dissemination research.” [121]

### Explanation

Many authors may also examine previously published search strategies to develop the search strategies for their review. Sometimes, authors adapt or reuse these searches for different systematic reviews [122]. When basing a new search strategy on a published search strategy, it is appropriate to cite the original publication(s) consulted.

Search strategies differ from filters (Item 10) because search strategies are often developed for a specific project, not necessarily designed to be repeatedly used. Filters, on the other hand, are developed with the express purpose of reuse. Filters are often objectively derived, tested, and validated, whereas most search strategies published as part of systematic review or other evidence synthesis are “best guess,” relying on the expertise of the searcher and review team [107].

As in the example above, researchers may rely on multiple prior published searches to construct a new search for a novel review. Many times, researchers will use the same searches from a published systematic review to update the existing systematic review. In either case, it is helpful to the readers to understand whether major portions of a search are being adapted or reused.

#### Suggested location for reporting

Report any prior work consulted, adapted, or reused in the methods section. Include the copied and pasted search strategies used, including portions or the entirety of any prior work used or adapted for use, in the full search strategy (Item 8).

### Item 12. Updates

Report the methods used to update the search(es) (e.g., rerunning searches, email alerts).

### Examples

“Ovid Auto Alerts were set up to provide weekly updates of new literature until July 09, 2012.” [123]

**“**Two consecutive searches were conducted and limited by publication type and by date, first from January 1, 1990, to November 30, 2012, and again from December 1, 2012, to July 31, 2015, in an updated search…. The original search strategy was used to model the updated search from December 1, 2012, to July 31, 2015. The updated search strategy was consistent with the original search; however, changes were required in the ERIC database search because of a change in the ERIC search algorithm. Excluded publication types were identical to the initial search. To improve specificity, the updated search was limited to human participants.” [99]

### Explanation

The literature search is usually conducted at the initial stage of the production of a systematic review. As a consequence, the results of a search may be outdated before the review is published [124–126]. The last search in a review should be conducted ideally less than 6 months before publication [90, 92, 125]. For this reason, authors often update searches by *rerunning* (Table 2) the same search(es) or otherwise updating searches before the planned publication date. Updating searches differs from updating a systematic review, i.e., when the same or different authors or groups decide to redo a published systematic review to bring its findings up to date. If authors are updating a published systematic review, either authored by the same review team or another, Item 11 contains relevant guidance.

When reporting search updates, the extent of reporting depends on methods used and any changes that were made while updating the searches. If there are no changes in information sources and/or search *syntax* (Table 2), it is sufficient to indicate the date the last search was run in the methods section and in the supplementary materials. If there are any changes in information sources and/or search syntax, the changes should be indicated (e.g., different set of databases, changes in search syntax, date restrictions) in the methods section. Authors should explain why these changes were made. When there were changes in the search strategy syntax, the original and the updated searches should both be reported as described in Item 8.

If authors use email alerts or other methods to update searches, these methods can be briefly described by indicating the method used, the frequency of any updates, the name of the database(s) used, or other relevant information that will help readers understand how the authors conducted search updates. If deduplication methods are used as part of the search update process, these methods can be described using guidance in Item 16.

#### Suggested location for reporting

Report the methods used to update the searches in the methods section and the supplementary materials, as described above.

### Item 13. Dates of searches

For each search strategy, provide the date when the last search occurred.

### Example

“A comprehensive literature search was initially run on 26 February 2017 and then rerun on 5 February 2018….” [127]

### Explanation

Most literature databases are regularly updated with new citations as articles are published. Citations already in the database may also be updated once new information (such as indexing terms or citing articles) is available. As an example, MEDLINE added over 900,000 *indexed* citations (Table 2) in fiscal year 2018 [41]. In addition, the information gathered by databases (such as author affiliations in MEDLINE) can change over time. Because new citations are regularly being added, systematic review guidelines recommend updating searches throughout the writing process to ensure that all relevant articles are retrieved [6, 92].

It is necessary for authors to document the date when searches were executed, either the date the initial search was conducted, if only searched once, or the most recent date the search was rerun. This allows readers to evaluate the currency of each search and understand what literature the search could have potentially identified [125]. In addition, it supports reproducibility and updating by allowing other researchers to use date limits to view the same “slice” of the database that the original authors used or to update a systematic review by searching from the last time point searched.

#### Suggested location for reporting

Report the date of the last search of the primary information sources used in the abstract for optimal clarity for readers [128]. Report the time frame during which searches were conducted, the initial search date(s), and/or the last update search date(s) in the methods section. Report the initial and/or last update search date with each complete search strategy in the supplementary materials, as in the examples for Item 8.

### Item 14. Peer review

Describe any search peer review process.

### Example

“The strategies were peer reviewed by another senior information specialist prior to execution using the PRESS Checklist [1].” [129]

### Explanation

Peer reviewing search strategies is an increasingly valued component of search strategy development for systematic reviews. Expert guidance recommends taking this step to help increase the robustness of the search strategy [6, 74]. *Peer reviewing* (Table 2) searches is useful to help to guide and improve electronic search strategies. One of peer review’s main benefits is the reduction of errors [23, 130]. Peer review may also increase the number of relevant records found for inclusion in reviews, thus improving the overall quality of the systematic review [131].

Authors should consider using the Peer Review of Electronic Search Strategies (PRESS) Guideline statement, a practice guideline for literature search peer review outlining the major components important to review and the benefits of peer reviewing searches [1]. Authors should strongly consider having the search strategy peer reviewed by an experienced searcher, information specialist, or librarian [1, 131]. Though peer review may be conducted generally with publication of a protocol, for example, this item is designed to document search-specific peer review.

#### Suggested location for reporting

Describe the use of peer review in the methods section.

### Item 15. Total records

Document the total number of records identified from each database and other information sources.

### Examples

*Methods section*. “A total of 3251 citations were retrieved from the six databases and four grey literature websites.” [133]

**Fig. 1:**
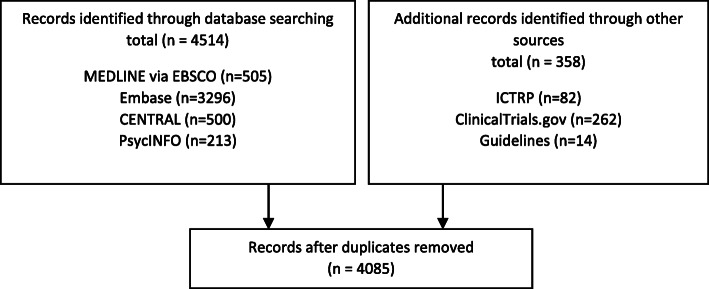
“Figure 1. PRISMA 2009 flow diagram” [132]

*Flow diagram*. Fig. 1.

### Explanation

Recording the flow of citations through the systematic review process is a key component of the PRISMA Statement [9, 35]. It is helpful to identify how many *records* (Table 2) were identified within each database and additional source. Readers can use this information to see whether databases or expert contacts constituted the majority of the records reviewed, for example. Knowing the number of records from each source also helps with reproducibility. If a reader tries to duplicate a search from a systematic review, one would expect to retrieve nearly the same results when limiting to the timeframe in the original review. If instead, the searcher locates a drastically different number of results than reported in the original review, this can be indicative of errors in the published search [23] or major changes to a database, both of which might be reasons to update a systematic review or view the systematic review’s results with skepticism.

#### Suggested location for reporting

Report the total number of references retrieved from all sources, including updates, in the results section. Report the total number of references from each database and information source in the supplementary materials. If space permits, report the total number of references from each database in the PRISMA flow diagram [35].

### Item 16. Deduplication

Describe the processes and any software used to deduplicate records from multiple database searches and other information sources.

### Example

“Duplicates were removed by the librarians (LP, PJE), using EndNote's duplicate identification strategy and then manually.” [134]

### Explanation

Databases contain significant overlap in content. When searching in multiple databases and additional information sources, as is necessary for a systematic review, authors often employ a variety of techniques to reduce the number of duplicates within their results prior to screening [135–138]. Techniques vary in their efficacy, sensitivity, and *specificity* (Table 2) [136, 138]. Knowing which method is used enables readers to evaluate the process and understand to what extent these techniques may have removed false positive duplicates [138]. Authors should describe and cite any software or technique used, when applicable. If duplicates were removed manually, authors should include a description.

#### Suggested location for reporting

Report any deduplication method used in the methods section. The total number of references after deduplication should be reported in the PRISMA flow diagram [35].

## Part 5. Discussion and conclusions

The PRISMA-S extension is designed to be used in conjunction with PRISMA 2020 [35] and PRISMA extensions including PRISMA-P for protocols [36], PRISMA-ScR for scoping reviews [139], the PRISMA Network Meta-analyses statement [140], and PRISMA-IPD for systematic reviews using individual patient data [141]. It may also be used with other reporting guidelines that relate to systematic reviews and related review types, such as RepOrting standards for Systematic Evidence Syntheses (ROSES) [142]. It provides additional guidance for systematic review teams, information specialists, librarians, and other researchers whose work contains a literature search as a component of the research methods. Though its origins are in the biomedical fields, PRISMA-S is flexible enough to be applied in all disciplines that use method-driven literature searching. Ultimately, PRISMA-S attempts to give systematic review teams a framework that helps ensure transparency and maximum reproducibility of the search component of their review.

PRISMA-S is intended to capture and provide specific guidance for reporting the most common methods used in systematic reviews today. As new methods and information sources are adopted, authors may need to adjust their reporting methods to accommodate new processes. Currently, PRISMA-S does not address using text mining or text analysis methods to create the search, for example, though this is an increasingly common way for information specialists to develop robust and objective search strategies [143–145]. Likewise, PRISMA-S does not require that decisions about the rationale behind choices in search terms and search construction be recorded, though this provides readers a great deal of insight. In the future, methods and rationales used to create search strategies may become more important for reproducibility.

PRISMA-S offers extensive guidance for many different types of information source and methods, many of them not described in detail in other reporting guidelines relating to literature searching. This includes detailed information on reporting study registry searches, web searches, multi-database searches, and updates. PRISMA-S can help authors report all components of their search, hopefully making the reporting process easier. As a note, PRISMA-S provides guidance on transparent reporting to authors and is not intended as a tool to either guide conduct of a systematic review or to evaluate the quality of a search or a systematic review.

The PRISMA-S checklist is available for download in Word and PDF formats from the PRISMA Statement web site [37]. The checklist should be used together with its Explanation & Elaboration documentation to provide authors with guidance for the complexities of different types of information sources and methods.

We intend to work with systematic review and information specialist organizations to broadly disseminate PRISMA-S and encourage its adoption by journals. In addition, we plan to host a series of webinars discussing how to use PRISMA-S most effectively. These webinars will also be available for later viewing and will serve as a community resource.

We hope that journal editors will recommend authors of systematic reviews and other related reviews to use PRISMA-S and submit a PRISMA-S checklist with their manuscripts. We also hope that journal editors will encourage more stringent peer review of systematic review searches to ensure greater transparency and reproducibility within the review literature.

## Supplementary Information

**Additional file 1.**

## Data Availability

All data is available via the PRISMA-S PRISMA Search Reporting Extension OSF site (10.17605/OSF.IO/YGN9W) [32]. This includes all data relating to item development, survey instruments, data from the Delphi surveys, and consent documents.

## Abbreviations

DOI: Digital object identifier
PRESS: Peer Review of Electronic Search Strategies
PRISMA: Preferred Reporting Items for Systematic reviews and Meta-Analyses
PRISMA-IPD: PRISMA for individual patient data
PRISMA-P: PRISMA for systematic review protocols
PRISMA-ScR: PRISMA for scoping reviews
ROSES: RepOrting standards for Systematic Evidence Syntheses
