# Supplementary material for: PRISMA-S: an extension to the PRISMA Statement for Reporting Literature Searches in Systematic Reviews
Source: Syst Rev. 2021 Jan 26;10:39. doi: 10.1186/s13643-020-01542-z (PMC7839230; doi:10.1186/s13643-020-01542-z)
Supplement: Supplementary file 1 — Additional file 1. [file 13643_2020_1542_MOESM1_ESM.pdf]

# Additional file 1. PRISMA-S: An Extension to the PRISMA Statement for Reporting Literature Searches in Systematic Reviews

Melissa L. Rethlefsen (Corresponding Author), Shona Kirtley, Siw Waffenschmidt, Ana Patricia Ayala, David Moher, Matthew J. Page, Jonathan B. Koffel, PRISMA-S Group

## Contents

Searches ........ 2
Sources for Items ........ 4
Flow Chart ........ 8

## Searches

**Embase via Embase.com** (Search date: 2/19/16; 1122 results, post dedeup 1086)

2005:py OR 2006:py OR 2007:py OR 2008:py OR 2009:py OR 2010:py OR 2011:py OR 2012:py OR 2013:py OR 2014:py OR 2015:py OR 2016:py AND ((document* OR reporting OR reproduc* OR replica*) NEAR/3 (search* OR literature OR medline OR embase)):de,ab,ti AND ((system*ic NEXT/1 review*):de,ab,ti OR synthes*:de,ab,ti)

**LISTA via EBSCO** (Search Date: 2/19/16; 116 results; post dedup 111)

( ((document* OR reporting OR reproduc* OR replica*) N3 (search* OR literature OR medline OR embase)) ) AND ( (systematic n2 review*) OR meta* OR (literature N2 review*) )

**MEDLINE via Ovid** (Search Date: Feb 2016; post de-dup 495, 507 before (based on Sampson et al search[48]))

Database: Ovid MEDLINE(R) and Epub Ahead of Print, In-Process & Other Non-Indexed Citations and Daily
Search Strategy:
--------------------------------------------------------------------------------
1 search$.tw.
2 exp "Information Storage and Retrieval"/
3 exp Medical Informatics/
4 Data Compression/
5 2 or 3
6 5 not 4
7 1 and 6
8 quality control/ or Evaluation Studies/
9 "Reproducibility of Results"/
10 8 or 9
11 (quality adj2 assess$).tw.
12 10 or 11
13 (quality or evaluat$).tw.
14 (precision or recall or sensitivity or relevance or specificity).tw.
15 (performance adj2 (measur$ or indicat$ or assess$)).tw.
16 "Sensitivity and Specificity"/
17 (error$ or mistake$ or failure$ or inaccura$ or misspell$).tw.
18 (MeSH or (subject adj2 heading$) or (controlled adj2 vocabulary) or redundan$ or explod$ or explos$ or spell$ or Medline).tw.
19 truncat$.tw.
20 (truncat$ adj6 (protein$ or gene$)).mp.
21 19 not 20
22 exp Documentation/

23 exp Molecular Sequence Data/
24 22 not 23
25 18 or 21 or 24
26 Evidence-Based Medicine/
27 Meta-Analysis/
28 Review.pt.
29 exp "Review Literature as Topic"/
30 26 or 27 or 28 or 29
31 "Information Storage and Retrieval"/st [Standards]
32 30 and 31
33 14 or 15 or 16
34 7 and 12 and 33
35 7 and 33 and 25
36 13 and 33 and 25
37 cochrane database of systematic reviews.jn.
38 32 or 34 or 35 or 36
39 38 not 37
40 limit 39 to yr="2005 -Current"
41 (documentation or (document* adj3 search*) or reporting).mp.
42 (presentation and publication).ti.
43 (presentation and standard*).ti.
44 (presentation and guideline*).ti.
45 minimum information.ab,ti.
46 (research standard or research standards).ab,ti.
47 minimum quality level.ab,ti.
48 (responsible and research).ti.
49 (uniform* and guideline*).ti.
50 (author* and guideline*).ti.
51 (advice and author*).ti.
52 (contributor* and journal*).ti.
53 (contributor* and journal*).ab,ti.
54 (publication* and data).ti.
55 journal*.ab,ti. and submiss*.ti.
56 journal*.ab,ti. and submit*.ti.
57 (recommendation* and editor*).ab,ti.
58 instruction*.ab,ti. and author*.ti.
59 (publication* and practice*).ti.
60 or/42-59
61 40 and (41 or 60)
62 (systematic* or meta).mp.
63 61 and 62
64 limit 63 to yr=”2005 -Current”

## Sources for Items

To see which source we derived which item from, see the Items for Delphi 1 Raw Sorted Harmonized file available on the project's OSF site: DOI 10.17605/OSF.IO/YGN9W.


1. Health Technology Assessment Handbook. In: FB Kristensen; H Sigmund, editors. Copenhagen, Denmark: Danish Centre for Health Technology Assessment, National Board of Health; 2007.
2. Systematic reviews. CRD's guidance for undertaking reviews in health care. York, UK: Centre for Reviews and Dissemination, University of York; 2008.
3. Identifying the evidence: literature searching and evidence submission. In: Developing NICE guidelines: the manual (January 2016 Update). London, UK: National Institute for Health and Care Excellence; 2014.
4. Systematic literature searches for literature based submissions. In: Standards & guidelines for prescription medicines. Symonston, Australia: Therapeutic Goods Administration, Department of Health, Australian Government; 2014.
5. American Psychological Association. APPENDIX. Journal Article Reporting Standards (JARS), Meta-Analysis Reporting Standards (MARS), and Flow of Participants Through Each Stage of an Experiment or Quasi-Experiment. In: Publication Manual of the American Psychological Association. Washington, DC: American Psychological Association; 2010; p. 245-53.
6. Atkinson KM, Koenka AC, Sanchez CE, Moshontz H, Cooper H. Reporting standards for literature searches and report inclusion criteria: making research syntheses more transparent and easy to replicate. Res Synth Methods. 2015 Mar;6(1):87-95. DOI: 10.1002/jrsm.1127.
7. Balshem H, Stevens A, Ansari M, Norris S, Kansagara D, Shamliyan T, Chou R, Chung M, Moher D, Dickersin K. Finding Grey Literature Evidence and Assessing for Outcome and Analysis Reporting Biases When Comparing Medical Interventions: AHRQ and the Effective Health Care Program. In Methods Guide for Effectiveness and Comparative Effectiveness Reviews, AHRQ Methods for Effective Health Care. Rockville (MD): Agency for Healthcare Research and Quality (US); 2008.
8. Beller EM, Glasziou PP, Altman DG, Hopewell S, Bastian H, Chalmers I, Gotzsche PC, Lasserson T, Tovey D. PRISMA for Abstracts: reporting systematic reviews in journal and conference abstracts. PLoS Med. 2013;10(4):e1001419. DOI: 10.1371/journal.pmed.1001419.
9. Boell SK, Cecez-Kecmanovic D. On being 'systematic' in literature reviews in IS. Journal of Information Technology (Palgrave Macmillan). 2015;30(2):161-73. DOI: 10.1057/jit.2014.26.
10. Briscoe S. Web searching for systematic reviews: a case study of reporting standards in the UK Health Technology Assessment programme. BMC Res Notes. 2015 Apr 16;8:153. DOI: 10.1186/s13104-015-1079-y.
11. Brouwers MC, Kho ME, Browman GP, Burgers JS, Cluzeau F, Feder G, Fervers B, Graham ID, Grimshaw J, Hanna SE, Littlejohns P, Makarski J, Zitzelsberger L. AGREE II: advancing guideline development, reporting and evaluation in health care. CMAJ. 2010 Dec 14;182(18):E839-42. DOI: 10.1503/cmaj.090449.
12. Chambers JD, Naci H, Wouters OJ, Pyo J, Gunjal S, Kennedy IR, Hoey MG, Winn A, Neumann PJ. An assessment of the methodological quality of published network meta-analyses: a systematic review. PloS one. 2015;10(4):e0121715. DOI:

13. Chen M, Xiao Y, Liu Y, Peng Y, He J, Zhang Y, Du L. The quality analysis of literature retrievals of systematic reviews for traditional Chinese medicine. Journal of Evidence-Based Medicine. 2015;8(1):42-52. DOI:
14. Cornell JE, Laine C. The science and art of deduction: complex systematic overviews. Ann Intern Med. 2008 May 20;148(10):786-8. DOI:
15. Craven J, Levay P. Recording database searches for systematic reviews - what is the value of adding a narrative to peer-review checklists? A case study of NICE interventional procedures guidance. Evid Based Libr Inf Pract. 2011;6(4):72-87. DOI:
16. de Vries RBM, Hooijmans CR, Langendam MW, van Luijk J, Leenaars M, Ritskes-Hoitinga M, Wever KE. A protocol format for the preparation, registration and publication of systematic reviews of animal intervention studies. Evidence-based Preclinical Medicine. 2015;2(1):1-9. DOI: 10.1002/ebm2.7.
17. Dudden RF, Protzko SL. The systematic review team: Contributions of the health sciences librarian. Medical Reference Services Quarterly. 2011;30(3):301-15. DOI:
18. Faggion Jr CM, Atieh MA, Park S. Search strategies in systematic reviews in periodontology and implant dentistry. Journal of Clinical Periodontology. 2013;40(9):883-8. DOI:
19. Fehrmann P. COMPUTER SEARCH REPORT CHECKLIST (CSRC). [Internet]. Kent, OH: University Libraries, Kent State University; 2008. <http://libguides.library.kent.edu/ld.php?content_id=15513000>.
20. Fehrmann P, Thomas J. Comprehensive computer searches and reporting in systematic reviews. Research Synthesis Methods. 2011;2(1):15-32. DOI: 10.1002/jrsm.31.
21. Flores-Mir C, Major MP, Major PW. Search and selection methodology of systematic reviews in orthodontics (2000-2004). American Journal of Orthodontics and Dentofacial Orthopedics. 2006;130(2):214-7. DOI:
22. Golder S, Loke Y, McIntosh HM. Poor reporting and inadequate searches were apparent in systematic reviews of adverse effects. Journal of clinical epidemiology. 2008;61(5):440-8. DOI:
23. Golder S, Loke YK, Zorzela L. Some improvements are apparent in identifying adverse effects in systematic reviews from 1994 to 2011. Journal of Clinical Epidemiology. 2013;66(3):253-60. DOI:
24. Golder S, Loke YK, Zorzela L. Comparison of search strategies in systematic reviews of adverse effects to other systematic reviews. Health Information & Libraries Journal. 2014;31(2):92-105. DOI: 10.1111/hir.12041.
25. Hammerstrøm K, Wade A, Jorgensen A-M. Searching for studies: A guide to information retrieval for Campbell Systematic Reviews; 2010. (Campbell Systematic Reviews 2010; Supplement 1).
26. Hausner E, Waffenschmidt S, Kaiser T, Simon M. Routine development of objectively derived search strategies. Syst Rev. 2012;1:19. DOI: DOI: 10.1186/2046-4053-1-19.
27. Higgins JPT, Green S. Cochrane Handbook for Systematic Reviews of Interventions. [Internet]. The Cochrane Collaboration; March 2011 [cited 18 December 2013]. <http://handbook.cochrane.org/>.
28. Ijaz S, Croucher RE, Marinho VCC. Systematic reviews of topical fluorides for dental caries: a review of reporting practice. Caries research. 2010;44(6):579-92. DOI:
29. Institute of Medicine (U.S.). Committee on Standards for Systematic Reviews of Comparative Effectiveness Research., Eden J. Finding what works in health care : standards for systematic reviews. Washington, D.C.: National Academies Press; 2011. xxii, 317 p. p.
30. Kable AK, Pich J, Maslin-Prothero SE. A structured approach to documenting a search strategy for publication: A 12 step guideline for authors. Nurse Education Today. 2012;32(8):878-86. DOI:
31. Kowalczyk N, Truluck C. Literature reviews and systematic reviews: What is the difference? Radiologic Technology. 2013;85(2):219-22. DOI:

32. Lefebvre C, Glanville J, Wieland LS, Coles B, Weightman AL. Methodological developments in searching for studies for systematic reviews: past, present and future? Syst Rev. 2013 Sep 25;2:78. DOI: 10.1186/2046-4053-2-78.
33. Liberati A, Altman DG, Tetzlaff J, Mulrow C, Gotzsche PC, Ioannidis JP, Clarke M, Devereaux PJ, Kleijnen J, Moher D. The PRISMA statement for reporting systematic reviews and meta-analyses of studies that evaluate healthcare interventions: explanation and elaboration. BMJ. 2009 Jul 21;339:b2700. DOI: 10.1136/bmj.b2700.
34. Maggio LA, Tannery NH, Kanter SL. Reproducibility of literature search reporting in medical education reviews. Academic Medicine. 2011;86(8):1049-54. DOI:
35. Major MP, Major PW, Flores-Mir C. An evaluation of search and selection methods used in dental systematic reviews published in English. Journal of the American Dental Association. 2006;137(9):1252-7. DOI:
36. Major MP, Major PW, Flores-Mir C. Benchmarking of reported search and selection methods of systematic reviews by dental speciality. Evidence-Based Dentistry. 2007;8(3):66-70. DOI:
37. Moher D, Liberati A, Tetzlaff J, Altman DG, Group P. Preferred reporting items for systematic reviews and meta-analyses: the PRISMA statement. BMJ. 2009 Jul 21;339:b2535. DOI: 10.1136/bmj.b2535.
38. Niederstadt C, Droste S. Reporting and presenting information retrieval processes: The need for optimizing common practice in health technology assessment. International Journal of Technology Assessment in Health Care. 2010;26(4):450-7. DOI:
39. O'Connor AM, Anderson KM, Goodell CK, Sargeant JM. Conducting Systematic Reviews of Intervention Questions I: Writing the Review Protocol, Formulating the Question and Searching the Literature. Zoonoses and Public Health. 2014;61(SUPPL1):28-38. DOI:
40. Peters JL, Sutton AJ, Jones DR, Rushton L, Abrams KR. A Systematic Review of Systematic Reviews and Meta-Analyses of Animal Experiments with Guidelines for Reporting. Journal of Environmental Science and Health, Part B. 2006 2006/10/01;41(7):1245-58. DOI: 10.1080/03601230600857130.
41. Publications APA, Communications Board Working Group on Journal Article Reporting S. Reporting standards for research in psychology: why do we need them? What might they be? Am Psychol. 2008 Dec;63(9):839-51. DOI: 10.1037/0003-066X.63.9.839.
42. Rader T, Mann M, Stansfield C, Cooper C, Sampson M. Methods for documenting systematic review searches: a discussion of common issues. Res Synth Methods. 2014 Jun;5(2):98-115. DOI: 10.1002/jrsm.1097.
43. Relevo R, Balshem H. Finding Evidence for Comparing Medical Interventions (AHRQ Publication No. 11-EHC021-EF). In: Methods Guide for Comparative Effectiveness Reviews., Series Finding Evidence for Comparing Medical Interventions (AHRQ Publication No. 11-EHC021-EF). Agency for Healthcare Research and Quality; 2011.
44. Rethlefsen ML, Farrell AM, Osterhaus Trzasko LC, Brigham TJ. Librarian co-authors correlated with higher quality reported search strategies in general internal medicine systematic reviews. J Clin Epidemiol. 2015;68(6):617-26. DOI:
45. Rethlefsen ML, Murad MH, Livingston EH. Engaging medical librarians to improve the quality of review articles. JAMA. 2014 September 10;312(10):999-1000. DOI:
46. Robinson KA, Chou R, Berkman ND, Newberry SJ, Fu R, Hartling L, Dryden D, Butler M, Foisy M, Anderson J, Motu'apuaka M, Relevo R, Guise JM, Chang S. Twelve recommendations for integrating existing systematic reviews into new reviews: EPC guidance. J Clin Epidemiol. 2016 Feb;70:38-44. DOI: 10.1016/j.jclinepi.2015.05.035.

47. Robinson KA, Whitlock EP, Oneil ME, Anderson JK, Hartling L, Dryden DM, Butler M, Newberry SJ, McPheeters M, Berkman ND, Lin JS, Chang S. Integration of existing systematic reviews into new reviews: Identification of guidance needs. Systematic Reviews. 2014;3(1)
48. Sampson M, McGowan J, Tetzlaff J, Cogo E, Moher D. No consensus exists on search reporting methods for systematic reviews. J Clin Epidemiol. 2008 Aug;61(8):748-54. DOI: 10.1016/j.jclinepi.2007.10.009.
49. Seehra J, Fleming PS, Polychronopoulou A, Pandis N. Reporting completeness of abstracts of systematic reviews published in leading dental specialty journals. European Journal of Oral Sciences. 2013;121(2):57-62. DOI:
50. Sena ES, Currie GL, McCann SK, Macleod MR, Howells DW. Systematic Reviews and Meta-Analysis of Preclinical Studies: Why Perform Them and How to Appraise Them Critically. Journal of Cerebral Blood Flow & Metabolism. 2014 12/11/received 01/21/accepted;34(5):737-42. DOI: 10.1038/jcbfm.2014.28.
51. Shea BJ, Grimshaw JM, Wells GA, Boers M, Andersson N, Hamel C, Porter AC, Tugwell P, Moher D, Bouter LM. Development of AMSTAR: a measurement tool to assess the methodological quality of systematic reviews. BMC Med Res Methodol. 2007;7:10. DOI: DOI: 10.1186/1471-2288-7-10.
52. Shemilt I, Simon A, Hollands GJ, Marteau TM, Ogilvie D, O'Mara-Eves A, Kelly MP, Thomas J. Pinpointing needles in giant haystacks: use of text mining to reduce impractical screening workload in extremely large scoping reviews. Research Synthesis Methods. 2014;5(1):31-49. DOI: 10.1002/jrsm.1093.
53. Stewart LA, Clarke M, Rovers M, Riley RD, Simmonds M, Stewart G, Tierney JF. Preferred Reporting Items for Systematic Review and Meta-Analyses of individual participant data: the PRISMA-IPD Statement. Jama. 2015 Apr 28;313(16):1657-65. DOI: 10.1001/jama.2015.3656.
54. Stovold E, Beecher D, Foxlee R, Noel-Storr A. Study flow diagrams in Cochrane systematic review updates: an adapted PRISMA flow diagram. Syst Rev. 2014 May 29;3:54. DOI: 10.1186/2046-4053-3-54.
55. Stroup DF, Berlin JA, Morton SC, Olkin I, Williamson GD, Rennie D, Moher D, Becker BJ, Sipe TA, Thacker SB. Meta-analysis of observational studies in epidemiology: a proposal for reporting. Meta-analysis Of Observational Studies in Epidemiology (MOOSE) group. JAMA. 2000 Apr 19;283(15):2008-12. DOI:
56. Thompson J, Davis J, Mazerolle L. A systematic method for search term selection in systematic reviews. Res Synth Methods. 2014 Jun;5(2):87-97. DOI: 10.1002/jrsm.1096.
57. Tong A, Flemming K, McInnes E, Oliver S, Craig J. Enhancing transparency in reporting the synthesis of qualitative research: ENTREQ. BMC Med Res Methodol. 2012 Nov 27;12:181. DOI: 10.1186/1471-2288-12-181.
58. Welch V, Petticrew M, Petkovic J, Moher D, Waters E, White H, Tugwell P. Extending the PRISMA statement to equity-focused systematic reviews (PRISMA-E 2012): explanation and elaboration. J Clin Epidemiol. 2016 Feb;70:68-89. DOI: 10.1016/j.jclinepi.2015.09.001.
59. Witkowski MA, Aldhouse N. Transparency and reproducibility of supplementary search methods in nice single technology appraisal manufacturer submissions. Value in Health. 2015;18(7):A721-A2. DOI:
60. Yoshii A, Plaut DA, McGraw KA, Anderson MJ, Wellik KE. Analysis of the reporting of search strategies in Cochrane systematic reviews. Journal of the Medical Library Association. 2009;97(1):21-9. DOI:
61. Zorzela L, Loke YK, Ioannidis JP, Golder S, Santaguida P, Altman DG, Moher D, Vohra S. PRISMA harms checklist: improving harms reporting in systematic reviews. Bmj. 2016 Feb 01;352:i157. DOI: 10.1136/bmj.i157.

## Flow Chart

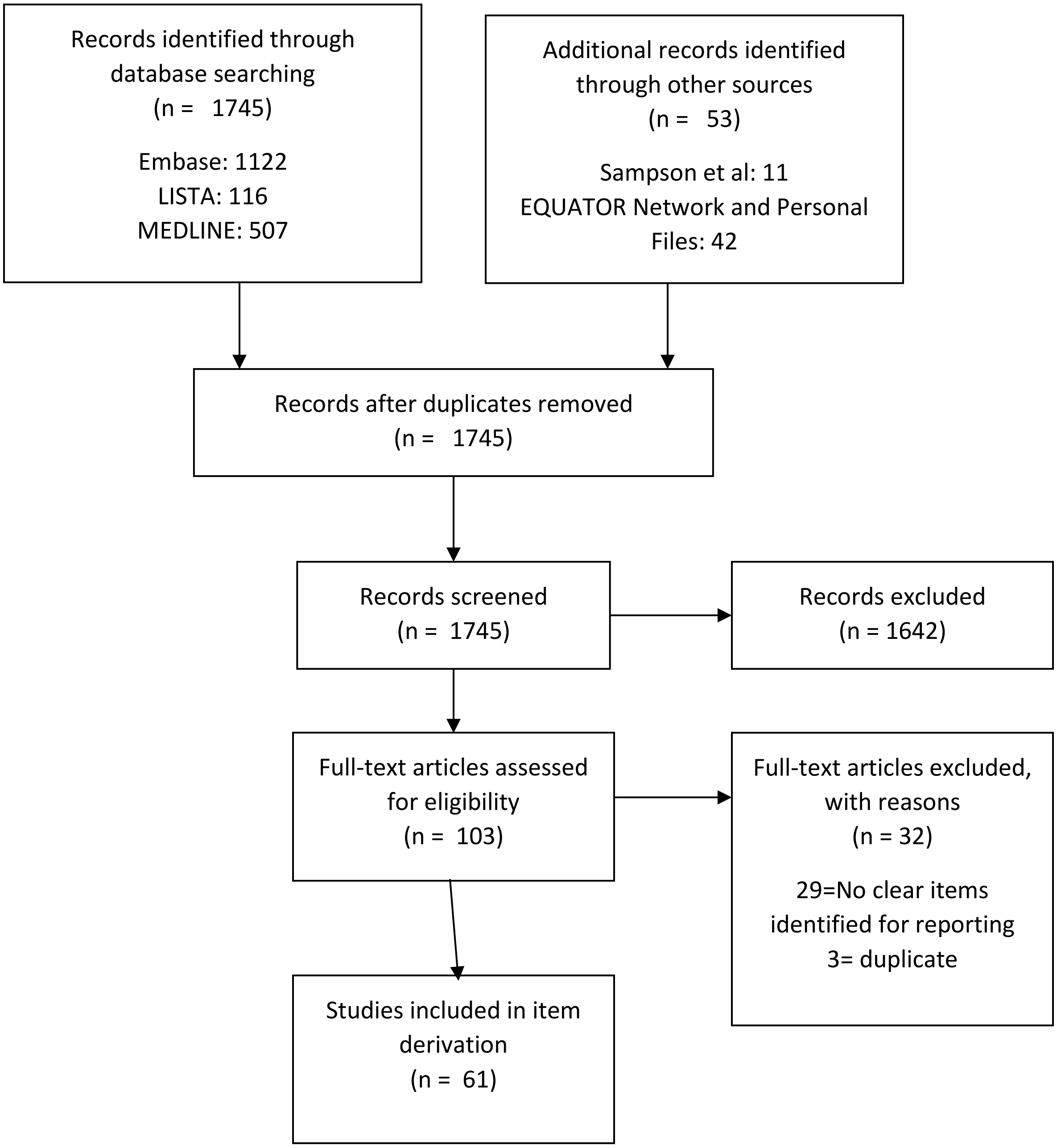

Records identified through database searching (n = 1745)
Embase: 1122
LISTA: 116
MEDLINE: 507
Additional records identified through other sources (n = 53)
Sampson et al: 11
EQUATOR Network and Personal Files: 42
Records after duplicates removed (n = 1745)
Records screened (n = 1745)
Records excluded (n = 1642)
Full-text articles assessed for eligibility (n = 103)
Full-text articles excluded, with reasons (n = 32)
29=No clear items identified for reporting
3= duplicate
Studies included in item derivation (n = 61)
